# Does plate waste matter?: A two-stage cluster survey to assess the household plate waste in the Philippines

**DOI:** 10.1186/s12889-022-14894-z

**Published:** 2023-01-04

**Authors:** Imelda Angeles-Agdeppa, Marvin Bangan Toledo, Jezreel Ann Taruc Zamora

**Affiliations:** Food and Nutrition Research Institute, Department of Science and Technology Compound, General Santos Avenue, Lower Bicutan, 1632 Taguig City, Philippines

**Keywords:** Plate waste, Food weighing, Food security, Food secure, Food insecure, Food consumption

## Abstract

**Background:**

Plate waste is an urgent global public health problem. Gaining better knowledge of the quantity and patterns of plate waste among households may give critical insights into resolving the greater problem of unnecessary plate waste. The study was conducted to determine the amount of plate wastage across food security levels of households and evaluate possible factors associated with plate waste.

**Methods:**

This investigation analyzed the data from the 2018 Expanded National Nutrition Survey. Food weighing, food inventory, and food recall were the methods used to collect household food consumption and plate waste. Household Food Insecurity Access Scale was used to identify levels of food security among households.

**Results:**

The present study has revealed that the average household plate waste of rice was 49.6 g ± 4.7; meat, fish, & poultry was 7.5 g ± 0.5; and vegetable was 6.7 g ± 0.3. Rice (58%), vegetables (18%), and meat (9%) were the top 3 most wasted foods among Filipino households. Test showed that there was a significant difference in the wastage of rice (*p* < *0.001*), corn (*p* < *0.001*), vegetables (*p* < *0.05*), fish (*p* < *0.001*), meat (*p* < *0.001*), and fats and oils (*p* = *0.001*) across household food security levels. Households with the highest consumption of rice was 1.24 (CI: 1.06 – 1.46) times more likely to have rice waste compared to those households with the lowest consumption. Households with a female household head was 0.82 (CI: 0.78 – 0.87) times less likely to have plate waste of rice and rice products compared to those with male household head. The odds of rice wasting of household in urban areas was 0.83 (CI: 0.77 – 0.89) times higher in contrast to rural areas. The odds of rice wasting was 1.38 (CI: 1.15 – 1.66) times higher for households in the rich quintile compared to the poorest quintile. Household with highest vegetable consumption were 3.56 (CI: 2.51 – 5.03) times more likely to have vegetable waste compared to those with the lowest consumption. Households with 5 members were 1.13 (CI: 1.01 – 1.27) times more likely to have vegetable waste. The odds of wasting vegetables was 1.50 (CI: 1.14–1.97) times greater among households in the richest quintile compared in the poorest quintile. Families with the highest fish, meat & poultry consumption was 1.38 (CI: 1.01 – 1.91) times more likely of having fish, meat & poultry waste than households with lowest consumption. Fish, meat, and poultry plate waste was 0.81 (CI: 0.68 – 0.96) times less likely in households with 5 members or less than in households with more than 5 members. Compared to households in the lowest quintile, those in the middle quintile were 1.55 (CI: 1.01 – 2.38) times more likely to throw away fish, meat, and poultry. The odds of wasting fish, meat, and poultry was 2.26 (CI: 1.35 – 3.79) times higher for those in the richest than those in the poorest quintile.

**Conclusions:**

Findings suggest that plate waste is indeed a public health problem that should be addressed. Future research studies should explore the nutrient losses that might stem from plate wastage in order to have a more accurate approach when it comes to the development of strategies and interventions aimed at reducing household plate waste.

## Background

Providing nutritious, safe, and affordable food for everybody on a sustainable basis is one of the world's biggest issues today, especially in Asia, where 515 million people are projected to be malnourished with Central and Southern Asia experiencing the highest rates of food insecurity [1]. Despite food insecurity, approximately one-third of all food produced for human consumption is reported to be wasted [2]. Plate waste is defined as edible portions of food which are left on the dining table or on the plates after the family has finished eating and are usually given to household pets or discarded [3–5].

The Sustainable Development Goal (SDG) 12.3 specifically targeted and aimed to halve plate waste and decrease food loss by 2030 [6]. Globally, the average quantity of food waste per capita per year is equivalent to 18 healthy meals, meaning it can provide one person with the dietary reference intake of 25 nutrients for 18 days on average [7], which can adversely affect one’s nutritional status. According to the UN Environment Programme's Food Waste Index Report 2021, 931 million tons of food was wasted in 2019, with 61% coming from households, 26% from food service, and 13% from retail [8].

In the Philippines, plate waste is closely linked to hunger incidence and threatened food security[9]. The Global Hunger Index of 2018 scored the Philippines 69 of 119 countries, with a serious level of hunger incidence [10]. Given that plate waste is mostly generated at home, the typical Filipino family generates 66.8g of plate waste each day which is 5.0g more than in 2015 [11]. Recently, the 2018 Expanded National Nutrition Survey (ENNS) Household Food Consumption Component, spearheaded by the Department of Science and Technology – Food and Nutrition Research Institute (DOST-FNRI), revealed that 48.0g of typical plate waste are composed of cereals and cereal products, 8.9g are fish, meat and poultry, 7.2g are vegetables, and the remaining 2.7g are other food categories [12].

The issue of plate waste has also received worldwide attention due to its adverse environmental impacts and unfavorable economic consequences [13–15]. Resource losses are made during the production, processing, storage, distribution, and consumption stages of food [5]. From an environmental perspective, substantial environmental load occurs throughout the food supply chain [16, 17]. Food loss and waste amount to a major squandering of resources, which includes land, water, energy, labor, and capital, all of which produce their own amounts of greenhouse gas emissions which then contribute to global warming and climate change [18]. Regarding food groups that are frequently being wasted, the 2013 Food and Agriculture Organization (FAO) analysis on global food waste and the environment reported that cereal wastage is a major issue [19]. Rice is a major contributor to this problem because of its high carbon intensity and high wastage, meat waste has a significant influence on the environment in terms of land use and carbon footprint, and wasted vegetables have a large carbon impact [7]. Cereals, meat, and sugar are three of the most significant food groups that have an adverse influence on the environment [7]. Reducing plate waste is hence beneficial for economic development and pivotal for global sustainability, as it relates to water consumption, land use, and greenhouse gas emissions [14, 20].

Few studies on plate waste have been published locally. A study conducted across Katipunan Avenue (Loyola Height), Philippines showed that both dining venues and the customers who accompany restaurants play a significant role in the development of plate waste in our society [21]. In Ilocos Norte, Philippines, factors such as lack of infrastructure, inadequate training, and information dissemination deter respondents from participating in food waste reduction and recycling [22]. Household size, monthly income, and planning were found to specifically influence food waste management [22]. Initiatives have also emerged in recent years towards reducing plate waste in the Philippines. Last 2013, the Philippine Rice Research Institute (PRRI) launched the "Be Riceponsible Campaign" aiming towards reducing rice wastage and Senate Bill 1863 "Anti-Rice Wastage Act of 2013" was filed [23]. Currently also being debated is the proposed “Zero Food Waste Act” that mandates the state to develop a system to redistribute surplus edible food from restaurants, fast food chains, hotels and other food establishments to people who have less access to food [24].

Therefore, gaining a better knowledge regarding plate waste in household settings may give critical insights into resolving the greater problem of unnecessary plate waste. Plate waste management in the society is an important and urgent research issue, whether it is connected to enhancing nutrition or minimizing environmental or economic implications. Hence, this study was conducted with the aim to determine the amount of plate waste across food security levels among Filipino households and to evaluate possible factors associated with plate waste. This is also the first local study to evaluate plate waste among Filipinos in a household setting.

## Methods

### Study design and population

The study utilized the data from 20, 151 Filipino household who participated in the 2018 Expanded National Nutrition Survey (ENNS) as secondary data. The 2018 ENNS is a cross-sectional, population-based survey that characterizes the health and nutritional status of the Filipino population which was conducted by the Department of Science and Technology—Food and Nutrition Research Institute (DOST-FNRI) from February – December 2018. The ENNS is a three-year rolling survey that collected data from 2018 to 2020. The 2018 ENNS adopted the 2013 Master Sample design of the Philippine Statistics Authority (PSA) for household-based surveys which is a two-stage cluster sampling design with barangays/Enumeration Areas (EAs) or group of adjacent small barangays/EAs as the primary sampling units (PSUs) with about 100–400 households, followed by the selection of secondary sampling units composed of housing units/households. The 2018 ENNS was designed to represent estimates in the national and provincial level. 16 replicates were designed for the provincial estimate and since the present study focused in national level estimates, this was only considered. The Ethics Committee of DOST-FNRI approved the survey protocol and data collection instruments. All surveyed households provided informed consent prior to participation. The detailed methodology of the sampling design of the survey can be found elsewhere [3].

### Data collection

Household dietary data including food cost, household plate waste, household food security level, and socioeconomic and demographic variables were extracted in the 2018 ENNS database.

### Household dietary consumption

Registered Nutritionist-Dietitian used a digital measuring scale (Sartorius AZ4101 Digital Dietary Balance) to weigh household food items. All food prepared and served to the household for the day was weighed before cooking or in its raw state. Plate waste, given-out food, and leftover food were also weighed in order to determine the actual weight of the food consumed. Non-perishable items that could be used during the measuring day, such as coffee, sugar, salt, cooking oil, and various condiments, were weighed at the beginning and end of the day. Household food consumption was recorded in terms of kind and amount. The Registered Nutritionist-Dietitian validated food weighing by weighing similar food items consumed by the household members outside of the home. A 24-h food recall was conducted among household members via face-to-face interview wherein household members were asked to recall their food consumption. Most of the time, food recalled were in a cooked state. Other foods which were eaten raw were reported in their raw state. To determine the size of various food items consumed, devices such as wooden matchboxes, tablespoons, and plastic circles were utilized [3].

### Food cost

Food Cost is the cost of food spent by the household on the food weighing day and consumed or the price of the food item in a specific measure. It includes costs of home-produced food and given-in food during food weighing day which were imputed based on the prevailing market price. For costs of food items which cannot be recalled by the respondent, actual inquiry is done from where the food was purchased. The average exchange rate to US Dollar to Philippine Peso is 52.6614 pesos in 2018 [25]. We have categorized food items as "Bought (1)" or "Free (0)". “Bought” refers to the food items that has cost and “Free”, if the food has no cost (e.g. “am” is the water we get after boiling cleaned rice; lemon grass; common food items that grow or naturally grow in surroundings).

### Household plate waste

Plate waste refers to the edible portions of food which are left on the dining table or on the plates after the household has finished eating and are usually given to household pets or discarded [3–5]. Registered Nutritionist-Dietitian used a digital measuring scale (Sartorius AZ4101 Digital Dietary Balance) to weigh household plate waste after they finished eating. Plate waste values were subtracted from the initial weight of cooked foods to get the actual consumption.

### Household food security

The Household Food Insecurity Access Scale (HFIAS) [26], which is specifically a pre-tested questionnaire, was utilized in the present study to identify levels of food security among Filipino households. A licensed nutritionist-dietitian conducted the face-to-face interviews and administered the questionnaire to the study participants. The questions were based on the household's food intake during the previous month, followed by inquiries on how frequently the family unit encountered the circumstances. The tool was first introduced to DOST-FNRI in 2013 by the World Food Program, adapted from USAID FANTA Project. It is used to assess one of the dimensions of food security which is accessibility or food access The HFIAS categorizes food insecurity into four levels: food secure, mild, moderate, and severe.

Table 1 categorizes the types of food insecurity faced by households based on their frequency level. A food secure household does not encounter any of the circumstances or only has to worry about food on rare occasions. A family becomes slightly food insecure if it is occasionally or frequently concerned about food, and/or is unable to consume preferred meals, and/or seldom has to eat fewer diverse foods and/or to eat foods they dislike. A moderately food insecure household sacrifices food quality by eating a less varied diet and/or undesirable foods on a regular or irregular basis and begins to reduce the number of foods by reducing the meal portion or the number of meals on a regular or irregular basis, but it does not experience the three most severe conditions. A severely food insecure household often decreases the amount of food consumed and exhibits the three most severe symptoms (running out of food, going to sleep, being hungry, and not eating for the whole day). Any family experiencing any of the three severe situations is already classified as highly food insecure [26].

**Table 1:** Categories of food insecurity^**a**^

Situation(s) experienced in the past month	Frequency		
	**Rarely 1-2x**	**Sometimes 3-10x**	**Often > 10x**
1. Worry about food	Food Secure	Mild	Mild
2. Unable to eat preferred foods	Mild	Mild	Mild
3. Eat just a few kinds of foods	Mild	Moderate	Moderate
4. Eat foods they really do not want to eat	Mild	Moderate	Moderate
5. Eat a smaller meal	Moderate	Moderate	Severe
6. Eat fewer meals in a day	Moderate	Moderate	Severe
7. No food of any kind in the household	Severe	Severe	Severe
8. Go to sleep hungry	Severe	Severe	Severe
9. Go a whole day and night without eating	Severe	Severe	Severe

^**a**^Household food insecurity access scale indicator guide, V.3

### Socioeconomic and demographic variables

Data on family economic status (wealth status), household size, place of household residence, age (years) and sex of the household head, educational level and occupation level of the family head were included in the study. The wealth index of Filipino households was determined through principal component analysis (PCA) which was based on variables such as household characteristics, household assets, infrastructure factors, and utility access. Scores were designated to each of the household asset and then was used to categorize wealth quintiles as poorest, poorest, middle, rich, and richest. The in-depth methods of measurement and categorization were presented elsewhere [3].

### Data process

#### Consumers

Household consumer variable was generated by scoring one (1) if the household consumed at least 10 g of each food group (Food groups in Table 4) while zero (0) if it was less than 10 g. Food group consumption of each household was dichotomized in order to attain the assumption of the binary logistic regression that the dependent variable must be binary [27].

#### Households plate waste status

Household with plate waste (more than 0 g) were scored one (1) while zero (0) if they do not have plate waste.

### Statistical analysis

Stata 15 was used for all statistical analyses performed in this study (Stata Statistical Software, release 15, Stata Corporation 2017). Mean and standard errors of food consumption, plate waste, and food cost were estimated. Number and proportion of the households of socio-demographic characteristics across plate waste were also evaluated. Food preference was examined using the percentage consumer of the top 30 primarily eaten food items by plate waste and food security level. Percentage contribution of each food group (food group in Table 4) to the total plate waste was calculated by summing the total wasted food group (e.g. rice and rice product) divided by the total plate waste from all food multiplied by 100.

The data was log transform using log (y + 0.01) to the plate waste data to take into account the zero (0) values in the datasets 0 [28]. However, the assumption of normality and equality of variance were still not met. Thus, a Kruskal–Wallis H test [29] was conducted to determine if plate waste amount was different across food security level. Test for independence using Pearson Chi-square statistics was conducted to determine which among the household characteristics was associated to food wasting, specifically among top 3 wasted food such as rice and rice product, all vegetables, and meat, fish, & poultry food group.

Multivariable logistic regression analysis was applied to determine the significant factors of food wasting, specifically among top 3 wasted food such as rice and rice product, all vegetables, and meat, fish, & poultry food group [30–32]. The odds ratios (OR) and 95% confidence intervals were presented in this study. Variables included in the regression model were household size, place of residence, wealth quintile (SES), household head’s age, sex, and educational level, household food security level and portion size. The portion size indicates the actual consumption of the households of rice, vegetables, and meat which was group into tertiles using the –xtile- command in Stata. Specifically, household were group into three with different level of consumption of each food item. Tertile 1 characterized as the lowest consumption, Tertile 2 as the intermediate consumption, and Tertile 3 as the highest consumption. All analyses set the significance level α at 0.05. All analyses were accounted for the sampling weights to account the complex survey design and to reflect nationally representative results. In STATA 15, the command -svy- was used to account for the complexity of the survey design when generating estimates such as mean, standard errors, and percentages, as well as when performing the Pearson's chi-square test and multivariable logistic regression. The -svy- fits statistical models for complex survey data by adjusting the results of a command for survey settings identified by -svyset- [33].

## Results

### Descriptive

Table 2 shows that the majority of Filipino household heads are males (77%) of which 44% are aged 30–49 and 40% are aged 50–69. The majority of family heads (44%) had at least a high school diploma and an elementary education. The majority of families (66%) have five or fewer family members. Filipino households had an average of 5 family members. More than half of the households (56%) were in rural areas, while 44% were in urban areas. Twenty-one percent (21%) of households were classified as being in the poorest quintile, 22% in the poor quintile, 21% in the middle quintiles, 19% in the rich quintiles, and 16% in the richest quintile. Only about half of the families (45%) were food secure, while 12% experienced mild food insecurity, three out of every ten households (30%) experience moderate food insecurity, and 13% experience severe food insecurity.

**Table 2:** Characteristics of Filipino households *n* = 20,151

Characteristics	Percentage ± Standard error
**Household head**	
**Age group**	
18–29 years old	6.4 ± 0.29
30–49 years old	44.5 ± 0.50
50–69 years old	39.6 ± 0.38
70 + years old	9.5 ± 1 0.63
**Sex**	
Male	76.6 ± 0.12
Female	23.4 ± 1.12
**Educational attainment**	
No grade completed	2.1 ± 0.39
At least elementary level	35.3 ± 1.64
At least high school level	43.8 ± 0.77
At least college level	18.5 ± 0.89
Others (SPED, Arabic, etc.)	0.3 ± 0.15
**Household size**	
≤ 5 members	66 ± 1.25
> 5 members	34 ± 1.25
**Type of residence**	
Rural	56.4 ± 7.21
Urban	43.6 ± 7.21
**Wealth quintile**	
Poorest	21.2 ± 2
Poor	22.4 ± 1.85
Middle	20.6 ± 0.74
Rich	19.3 ± 1.68
Richest	16.5 ± 1.69
**Household food security**	
Food secure	44.8 ± 1.14
Mild food insecure	12.6 ± 0.42
Moderate food insecure	29.7 ± 1.08
Severe food insecure	12.9 ± 0.84

**Fig. 1:**
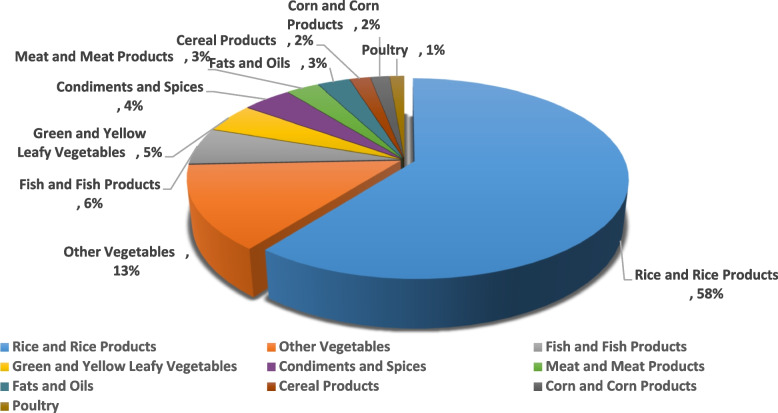
Percentage contribution of the top 10 mostly wasted food groups

Figure 1 shows that rice was the most wasted food item among Filipino households, accounting for 58% of total plate waste, followed by other vegetables (13%), fish and fish products (6%), and green and yellow leafy vegetables (5%), with a minor contribution from condiments and spices (4%), meat and meat products (3%), fats and oils (3%), cereal products (2%), corn and corn products (2%), and poultry (1%). Other food categories have a slightly lower contribution (< 1%).

### Food preference and plate waste

Table 3 summarized the top 30 commonly consumed food items among Filipino household. A combination of rice-vegetables-fish/meat diet remained the usual meal pattern observed in the table. Majority of the food items were bought. Rice was the top 1 (97%) commonly consumed food with an average of 1062 g per household while 37% of the household had rice waste with an average of 48 g per household. Almost all households (97%) were buying rice with average cost of 53 pesos. Other commonly consumed food items appear to be not wasted by families.

**Table 3:** Top 30 commonly consumed food items and plate waste among Filipino households, 2018

Plate waste					
**Percentage of household**			**Mean ± Standard error**		
**Food items**	Consumer (%)	Plate Waste (%)	Average consumption (g)	Average Cost (Php) (Average Exchange rate during 2018 was $1 = 52.6614 pesos)	Average Plate Waste (g)
Rice, well-milled	97	37	1061.9 ± 5.2	52.6 ± 0.6	48 ± 0.8
Salt	68	0	17.9 ± 0.2	0.5 ± 0	0.1 ± 0
Coconut oil	62	1	48.4 ± 0.4	5.1 ± 0.1	0.6 ± 0
Onion	52	1	34.7 ± 0.3	4.8 ± 0.1	0.3 ± 0
Brown sugar	42	0	45.9 ± 0.6	2.8 ± 0.1	0 ± 0
Chicken egg	42	1	152.3 ± 1.1	22.9 ± 0.5	0.6 ± 0.1
Garlic	42	1	14.1 ± 0.2	2.8 ± 0	0.2 ± 0
3 in 1 coffee	40	0	52.7 ± 0.5	13.5 ± 0.2	0 ± 0
Soy sauce	34	0	40.1 ± 0.5	3 ± 0.2	0.2 ± 0
Sodium Glutamate	29	0	4.3 ± 0.1	1.3 ± 0	0 ± 0
Instant Coffee	28	0	6.9 ± 0.2	5.7 ± 0.3	0 ± 0
Tomato	21	1	100.9 ± 2	6.4 ± 0.2	1 ± 0.1
Powder seasoning mix	21	0	6.3 ± 0.1	3 ± 0	0 ± 0
Powdered milk	20	0	57.3 ± 0.8	21.3 ± 0.4	0 ± 0
Eggplant	17	1	181.9 ± 3.1	11.8 ± 0.3	2.3 ± 0.2
Ginger	16	4	16.6 ± 0.9	3.2 ± 0.1	4.3 ± 0.1
Horseradish leaves	15	1	49.4 ± 3	6.6 ± 0.2	0.6 ± 0.2
String bean pod	15	1	156.1 ± 0.3	10.1 ± 0.2	1.6 ± 0.2
Pork Boston butt	15	1	279.2 ± 5.8	58.6 ± 1.3	4.3 ± 0.5
Squash fruit	15	1	239.7 ± 4.8	12.2 ± 0.3	2.9 ± 0.3
Cane vinegar	14	0	41.3 ± 0.8	3.1 ± 0.2	0.2 ± 0.1
Canned sardines	14	0	178.3 ± 2	22.2 ± 0.3	1.1 ± 0.2
Palm oil	13	0	49.4 ± 0.9	5.2 ± 0.2	0.8 ± 0.3
Pandesal (Bread)	13	0	221.4 ± 3.3	23.8 ± 6.8	0.7 ± 0.2
Carrot	12	0	50.1 ± 1.4	7 ± 0.2	0.7 ± 0.1
Okra	12	0	83.9 ± 1.9	7.1 ± 0.2	0.9 ± 0.1
Iodized Salt	12	0	15.7 ± 0.4	0.5 ± 0	0.1 ± 0
Pork belly	11	0	245 ± 5.8	54.4 ± 1.4	2.6 ± 0.7
Coffee creamer	10	0	21.4 ± 0.4	6.7 ± 0.2	0 ± 0
Hotdog	10	0	170.1 ± 3.5	27.7 ± 0.7	0.3 ± 0.1

### Food security and plate waste

Table 4 showed that the average cereal and cereal product waste was 50 g and most of this were from rice with 49.6 g per household. Rice was mostly wasted food followed by fish, meat, and poultry with an average of 8 g and mostly coming from fish and fish product with a mean of 4.5 g per household. Next top wasted food was vegetable with an average of 7 g per household and mostly coming from non-leafy/other vegetables with average of 5 g per family. Fruit waste has an average of 0.5 g, starchy roots and tubers has 0.4 g, fats and oil has 0.4 g, and other food groups showed minimal average plate wastage. Test showed that there were statistically significant differences in the amount of plate waste coming from rice (*p* =  < 0.001), corn (*p* =  < 0.001), vegetables (*p* = 0.005), fish (*p* =  < 0.001), meat (*p* =  < 0.001), and fats and oils (*p* = 0.001) across household food security level. Major number of households has no meat waste (92%) whereas 8% had.

**Table 4:** Mean ± standard error of plate waste (in grams) by food security level

Food security						
**Food groups**	**All**	**Food secure**	**Mild food insecure**	**Moderate food insecure**	**Severely food insecure**	***p*-value**
**Cereals and cereal products intake**	50.2 ± 4.7	53.3 ± 4.8	59.15 ± 7.64	47.5 ± 4.3	38.6 ± 4.3	< 0.001*
Rice and products intake	49.6 ± 4.7	52.7 ± 4.8	58.67 ± 7.66	47 ± 4.4	37.9 ± 4.4	< 0.001*
Corn and products intake	0.2 ± 0.1	0.3 ± 0.1	0.18 ± 0.1	0.2 ± 0.1	0.5 ± 0.3	< 0.001
Cereal products intake	0.3 ± 0.05	0.3 ± 0.1	0.3 ± 0.12	0.3 ± 0.1	0.2 ± 0.1	0.332
**Starchy roots and tubers intake**	0.4 ± 0.1	0.5 ± 0.1	0.38 ± 0.17	0.2 ± 0.1	0.5 ± 0.2	0.985
**Sugar and syrups intake**	0.004 ± 0.002	0.004 ± 0.003	0	0.01 ± 0.005	0	0.402
**Dried beans, nuts and seeds intake**	0.3 ± 0.04	0.4 ± 0.1	0.3 ± 0.09	0.2 ± 0.1	0.1 ± 0.1	0.102
**Vegetables intake**	6.7 ± 0.3	6.9 ± 0.4	6.81 ± 0.64	6.8 ± 0.7	6.2 ± 0.8	0.005*
Green leafy and yellow intake	1.6 ± 0.1	1.6 ± 0.2	1.91 ± 0.28	1.7 ± 0.2	1.3 ± 0.1	0.799
Other vegetables intake	5.1 ± 0.3	5.2 ± 0.4	4.9 ± 0.6	5.1 ± 0.6	4.9 ± 0.8	0.001*
**Fruits intake**	0.4 ± 0.1	0.6 ± 0.2	0.24 ± 0.11	0.3 ± 0.1	0.6 ± 0.4	0.074
Vitamin C-rich fruits intake	0.05 ± 0.02	0.1 ± 0.04	0	0.02 ± 0.02	0	0.05
Other fruits intake	0.4 ± 0.1	0.5 ± 0.2	0.24 ± 0.11	0.3 ± 0.1	0.6 ± 0.4	0.244
**Fish, meat and poultry intake**	7.6 ± 0.5	11.2 ± 0.8	7.23 ± 0.93	4.4 ± 0.4	4.5 ± 0.6	< 0.001*
Fish and products intake	4.5 ± 0.3	6.4 ± 0.5	4.45 ± 0.62	2.7 ± 0.3	3.2 ± 0.5	< 0.001
Meat and products intake	2 ± 0.2	3.3 ± 0.4	1.85 ± 0.35	0.9 ± 0.1	0.8 ± 0.2	< 0.001*
Poultry intake	1.1 ± 0.2	1.5 ± 0.1	0.93 ± 0.33	0.8 ± 0.1	0.5 ± 0.3	< 0.001*
**Eggs intake**	0.2 ± 0.04	0.4 ± 0.1	0.19 ± 0.06	0.2 ± 0.04	0.1 ± 0.1	0.492
**Milk and milk products intake**	0.03 ± 0.01	0.02 ± 0.01	0.01 ± 0.01	0.1 ± 0.03	0	0.253
Whole milk intake	0.03 ± 0.01	0.01 ± 0.01	0.01 ± 0.01	0.1 ± 0.03	0	0.210
Milk products intake	0.004 ± 0.002	0.005 ± 0.005	0	0.01 ± 0.01	0	-
Human milk intake	-	-	-	-	-	-
**Fats and oils intake**	0.4 ± 0.07	0.6 ± 0.1	0.3 ± 0.1	0.3 ± 0.1	0.2 ± 0.1	0.001*
**Miscellaneous intake**	0.5 ± 0.1	0.6 ± 0.1	0.6 ± 0.22	0.5 ± 0.1	0.5 ± 0.2	0.114
Beverages intake	0.01 ± 0.003	0.01 ± 0.01	0.01 ± 0	0.004 ± 0.002	0 ± 0	0.364
Condiments and spices intake	0.5 ± 0.1	0.5 ± 0.1	0.6 ± 0.2	0.5 ± 0.1	0.5 ± 0.2	0.123
Others intake	0.02 ± 0.01	0.05 ± 0.03	0.01 ± 0.01	0.005 ± 0.004	0	-

^*^significant at 5% level of significance using Kruskal–Wallis H test

### Socio-demographic and plate waste

Table 5 shows the top mostly wasted food across socio-demographic characteristics of the households. About 62% of the Filipino household has no rice waste while approximately two out of five (39%) families had rice waste. Majority (89%) of the household has no vegetable waste while 11% had. Major number of households has no meat waste (92%) whereas 8% wasted meat. Rice wasting is less common in households with a younger household head (5%), while it is more common in households with a household head aged 50–69. (42%). Rice waste is more mutual in households with a male household head (79%). When compared families with no rice waste, families with a household head who has at least an elementary education (38%) tend to have a higher rate of rice wasting, while households with a household head who has at least a college degree appear to have a lower incidence of rice wasting (17%). Rice appears to be wasted more in families with five or more members (36%). Rice was wasted at a higher rate (64%) in rural homes. Food insecure households squander more rice in the mild (14%) and moderate (31%) categories. Rice waste was associated to the age, sex, educational level, household size, type of residence, and food security level of the household head, according to the Pearson X2 test.

**Table 5:** Univariate association between social and demographic factors and plate wastage

Top 3 Wasted food									
**Characteristics**	**Rice**		***p*-value**	**Vegetable**		***p*-value**	**Fish, meat, & poultry**		***p*-value**
	No rice waste	Had rice waste		No vegetable waste	Had vegetable waste		No meat waste	Had meat waste	
	*n (Column %)*	*n (Column %)*		*n (Column %)*	*n (Column %)*		*n (Column %)*	*n (Column %)*	
**All**	12,373 (61.4)	7,778 (38.6)		17,955 (89.1)	2,196 (10.9)		18,571 (92.1)	1,580 (7.8)	
**Household head characteristics**									
**Age group**									
18–29 years old	876 (7.1)	416 5.3)	0.005*	1,183 (6.6)	109 (5)	0.112^NS^	1,213 (6.5)	79 (5)	0.217^NS^
30–49 years old	5,612 (45.4)	3,349 (43)		8,017 (44.6)	944 (43)		8,303 (44.7)	658 (41.7)	
50–69 years old	4,713 (38.1)	3,269 (42)		7,029 (39.1)	953 (43.4)		7,291 (39.3)	692 (43.8)	
70 + years old	1173 (9.5)	744 (9.6)		1,725(9.6)	190 (8.7)		1,766 (9.5)	150 (9.5)	
**Sex**									
Male	9,292 (75.1)	6,143 (79)	< 0.001*	13,724 (76.4)	1,711 (77.9)	0.419^NS^	14,252 (76.7)	1,184(75)	0.256^NS^
Female	3,081 (24.9)	1,635 (21)		4,230 (23.6)	486 (22.1)		4,320 (23.3)	396 (25)	
**Educational attainment**									
No Grade Completed	265 (2.1)	151 (1.9)	0.013*	370 (2.1)	46 (2.1)	0.192^NS^	403 (2.2)	12 (0.8)	0.001*
At Least Elementary Level	4,181 (33.8)	2,925 (37.6)		6,417 (35.7)	688 (35.7)		6,661 (35.9)	445 (28.1)	
At Least High School Level	5,451 (44.1)	3,377 (43.4)		7,799 (43.4)	1,028 (46.8)		8,077 (43.5)	752 (47.6)	
At Least College Level	2,419 (19.5)	1,311 (16.9)		3,302 (18.4)	429 (19.5)		3,361 (18.1)	369 (23.3)	
Others (SPED, Arabic, etc.)	57 (0.5)	14 (0.2)		65 (0.4)	5 (0.2)		69 (0.4)	2 (0.1)	
**Household size**									
≤ 5 members	8,331 (67.3)	4,966 (63.8)	0.003*	11,981 (66.7)	1,317(60)	< 0.001*	12,209 (65.7)	1,089 (68.9)	0.199^NS^
> 5 members	4,042 (32.7)	2,813 (36.2)		5,973 (33.3)	880 (40)		6,362 (34.3)	491 (31.1)	
**Type of residence**									
Rural	6,415 (51.8)	4,945 (63.6)	< 0.001*	10,160 (56.6)	1,200 (54.6)	0.527^NS^	10,599 (57.1)	761(48.2)	0.122^NS^
Urban	5,957 (48.2)	2,833 (36.2)		7,794 (43.4)	996 (45.4)		7,973 (42.9)	819 (51.8)	
**Wealth quintile**									
Poorest	2,602 (21)	1,679 (21.6)	0.507^NS^	3,903 (21.7)	379 (17.3)	0.005*	4,099 (22.1)	182 (11.5)	< 0.001*
Poor	2,748 (22.2)	1,770 (22.8)		4,065 (22.6)	453 (20.6)		4,240 (22.8)	278 (17.6)	
Middle	2,518 (20.3)	1,628 (20.9)		3,726 (20.7)	420 (19.1)		3,822 (20.6)	323 (20.5)	
Rich	2,378 (19.2)	1,510 (19.4)		3,399 (18.9)	489 (22.2)		3,486 (18.8)	403 (25.5)	
Richest	2,128 (17.2)	1,192 (15.3)		2,864 (15.9)	455 (20.7)		2,925 (15.7)	395 (25)	
**Household food security**									
Food Secure	5,761 (46.6)	3,273 (42.1)	0.006*	8,008 (44.6)	1,026 (46.7)	0.355^NS^	8,190 (44.1)	843 (53.4)	0.010*
Mild Food Insecure	1,402 (11.3)	1,131 (14.5)		2,243 (12.5)	290 (13.2)		2,329 (12.5)	205 (13)	
Moderate Food Insecure	3,560 (28.8)	2,435 (31.3)		5,368 (29.9)	626 (28.5)		5,603 (30.2)	391 (24.7)	
Severe Food Insecure	1,651 (13.3)	940 (12.1)		2,336 (13)	255 (11.6)		2,450 (13.2)	141 (8.9)	

*n (Column %)* were the weighted *n* and percentages of household

^*^significant at 5% level of significance using Chi-square test for association

Vegetable wasting was more common with families with five or more members (40%). Wasted vegetables were higher for wealthier households (rich quintile 22%, richest quintile 21%). Prevalence of vegetables wasting appears to be equal across other characteristics of the households. Test showed that vegetable wasting was associated to household size and wealth quintile.

Households with a household head who has at least a high school education (48%) or a college level (23%) had a greater rate of meat waste. The same was observed, meat waste was significantly higher in wealthy households (rich quintile 25%, richest quintile 25%). A household that is food secure wastes more meat (53%). The prevalence of meat wasting appears to be consistent across other household factors. Meat waste was shown to be associated to the educational level of the family head, wealth quintile, and food security.

### Factors associated to rice, vegetables, and meat wasted

In the multivariable logistic analysis in Table 6, regression model showed that the odds of wasting rice and rice products was 1.21 (95% Confidence Interval [CI]: 1.04 – 1.42) times higher among those household with intermediate consumption compared to those family with the lowest consumption of rice and rice products. Also, household with highest consumption of rice and rice products was 1.24 (1.06 – 1.46) times more expected to have plate waste compared to those family with the lowest consumption. The probability of a family with a female household head was 0.82 (0.78 – 0.87) times less likely to have plate waste of rice and rice products compared to those with male household head. The likelihoods of a household to have plate waste of rice and rice products in urban areas was 0.83 (0.77 – 0.89) times higher in contrast to rural areas. Family in the middle quintile was 1.19 (1.03 – 1.38) times more likely to waste rice and rice product compared to those in the poorest quintile. The odds of rice and rice product wasting was 1.38 (1.15 – 1.66) times higher for homes in the rich quintile compared to the poorest quintile.

**Table 6:** Factors associated to rice, vegetables, and meat wasting

Top 3 wasted food			
	**Rice**	**Vegetable**	**Fish, Meat, & Poultry**
**Factors**	Odds Ratio (95% CI)	Odds Ratio (95% CI)	Odds Ratio (95% CI)
**Household rice intake in tertiles** *(portion size)*			
Lowest consumption	Ref	-	-
Intermediate consumption	1.21 (1.04 – 1.42)*	-	-
Highest consumption	1.24 (1.06 – 1.46)*	-	-
**Household vegetable intake in tertiles** *(portion size)*			
Lowest consumption	-	Ref	-
Intermediate consumption	-	2.74 (1.99 – 3.80)**	-
Highest consumption	-	3.56 (2.51 – 5.03)**	-
**Household fish, meat, & poultry intake in tertiles** *(portion size)*			
Lowest consumption	-	-	Ref
Intermediate consumption	-	-	1.39 (1.14 – 1.70)*
Highest consumption	-	-	1.38 (1.01 – 1.91)*
**Household head**			
**Age**	1.01 (1—1.01)	1 (1 – 1.01)	1.01 (1 – 1.02)
**Sex**			
Male	Ref	Ref	Ref
Female	0.82 (0.78 – 0.87)**	0.92 (0.73 – 1.16)	1 (0.86 -1.16)
**Education**			
No grade completed	Ref	Ref	Ref
At least elementary level	1.27 (1.02 – 1.59)	0.80 (0.56 – 1.14)	1.58 (0.43 – 5.78)
At least high school level	1.26 (0.90 – 1.77)	0.94 (0.61—1.44)	1.84 (0.47 – 7.15)
At least college level	1.16 (0.96 – 1.41)	0.85 (0.43 – 1.66)	1.75 (0.47 – 6.44)
Others (SPED, Arabic, Others)	0.47 (0.24 – 0.93)	0.61 (0.31 – 1.20)	0.97 (0.12 – 7.76)
**Household size**			
< 5 members	Ref	Ref	Ref
≥ 5 member	1.1 (1.01 – 1.19)	1.13 (1.01 – 1.27)^a^	0.81 (0.68 – 0.96)^a^
**Urbanity**			
Rural	Ref	Ref	Ref
Urban	0.83 (0.77 – 0.89)**	1.02 (0.87 – 1.19)	1.05 (0.81 – 1.36)
**Wealth quintile**			
Poorest	Ref	Ref	Ref
Poor	1.12 (0.96 – 1.31)	1.13 (0.95 – 1.36)	1.30 (0.72 – 2.37)
Middle	1.19 (1.03 – 1.38)*	1.1 (0.96 – 1.27)	1.55 (1.01 – 2.38)*
Rich	1.38 (1.15 – 1.66)*	1.44 (1.23 – 1.69)*	2 (1.13 – 3.62)*
Richest	1.34 (1.1 – 1.68)	1.50 (1.14 – 1.97)*	2.26 (1.35 – 3.79)*
**Food security**			
Food secure	Ref	Ref	Ref
Mildly food insecure	1.30 (1 – 1.65)	1.1 (0.73 – 1.61)	1.06 (0.72 – 1.55)
Moderately food insecure	1.12 (0.96 – 1.31)	1.04 (0.83 – 1.31)	0.97 (0.70 – 1.35)
Severely food insecure	1.02 (0.82 – 1.25)	1.1 (0.81 – 1.41)	0.93 (0.62 – 1.39)

*significant  with *p*-value < 0.05, **significant with *p*-value < 0.001

For the characteristics affecting vegetable waste status, the chances of wasting vegetables were 2.74 (95% confidence interval [CI]: 1.99 – 3.80) times greater among households with intermediate vegetable intake compared to those with the lowest vegetable consumption. Additionally, households with the highest vegetable intake were 3.56 (2.51 – 5.03) times more likely to have plate waste than those with the lowest consumption. In comparison to households with > 5 people, families with 5 members were 1.13 (1.01 – 1.27) times more likely to have plate waste of rice and rice product. The probabilities of wasting vegetables were 1.44 (1.23–1.69) and 1.50 (1.14–1.97) times greater for households in the rich and richest quintiles, respectively, than for those in the lowest quintile.

Among the factors associated with fish, meat, and poultry waste, families with intermediate intake of fish, meat, and poultry had 1.39 (95% confidence interval [CI]: 1.99 – 3.80) times the likelihood of squandering these items than homes with low intake. The homes that consumed the most fish, meat & poultry also had a 1.38 (1.01 – 1.91) times higher chance of having plate waste than the ones that consumed the least of these items. Fish, meat, and poultry plate waste was 0.81 (0.68 – 0.96) times less common in homes with 5 members than in households with more than 5 members. Compared to families in the lowest quintile, those in the middle quintile were 1.55 (1.01 – 2.38) times more likely to throw away fish, meat, and poultry. The likelihood of wasting fish, meat, and poultry was 2.26 (1.35 – 3.79) times higher for those in the richest quintile and 2 (1.13 – 3.62) times greater for those in the rich quintiles than for those in the poorest.

## Discussion

The present study revealed that rice has the highest average household plate waste (49.6 g), followed by meat, fish, & poultry (7.5 g), and vegetable (6.7 g). These three food groups were also classified as the top 3 most wasted foods among Filipino households which is similar to a previous study conducted in China [34]. In particular, rice, which constitutes the highest contribution to plate waste, makes up the main bulk of the ordinary Filipino dining plate, contributing to the high carbohydrate intake compared to other nutrients [35]. As stated in a previous study, the availability of cheap food products such as rice has been reported to encourage overbuying and hoarding behaviors that increase the likelihood of plate waste [36]. In fact, even though Philippines is the world's biggest rice importer for several years, the International Rice Research Institute (IRRI) reported that at least $223 million of rice a year which is enough to feed 4.3 million people, is wasted [37].

This study also demonstrated that households with the highest rice consumption were more likely to have rice wastage compared to those households with the lowest consumption. Households with a female household head, located in urban areas, and categorized under rich quintile were found to be less likely to waste rice and rice products compared to their counterparts. The higher wastage on rice and rice products among households with a higher consumption could be explained that some habits related to plate waste include the preference for consuming freshly prepared rice instead of leftovers [38], yet this was not explored in the present study. Male headed households were also found to have wasted more food than female headed households in previous literature [39]. In terms of urbanity, a previous study hypothesized that urban households wasted more food than rural households due to higher income and the need to store food at home rather than harvesting it on demand which usually takes place in rural settings [40]. The present study also contributes to existing dearth of literature on rice in the consumption stage, specifically rice waste at the household context [41–43].

Households with the highest vegetable consumption were more likely to waste vegetables compared to those with the lowest consumption. This implies that more wastage is incurred with higher quantities purchased [44] and households are routinely purchasing large amounts of nutritious produce that they are not consuming [45]. Reasons for throwing away vegetables could primarily be spoilage after meal preparation since the shelf-life of vegetables is affected considerably by storage conditions and temperature [46, 47], thus over-purchasing could contribute to a higher amount of plate waste. From a nutritional standpoint, the incidence of wasted vegetables is especially alarming, given vegetables is a rich source of key essential nutrients [45]. Indeed, the study suggests that if households can successfully lower their plate waste generation by eating the vegetables they procure, they may improve the quality of their diets.

In terms of protein foods, this study reported that households with the highest fish, meat & poultry consumption were more likely to practice wastage than households with the lowest consumption. The odds of wasting fish, meat, and poultry were higher for those in the richest than for those in the poorest quintile. As reported by previous literature, health concerns are usually associated with the increased amount of highly perishable food wastage, such as meat and fish, due to the knowledge on the increased risks and consequences of consuming such products if spoiled [48].

Additionally, household size was also analyzed in the present study in relation to the odds of plate waste. Households composed of ≤ 5 members were found to have greater odds of wasting vegetables. This entails that plate waste decreases with household size, which could be interpreted as a reflection of scale economies in which quantity or diversity of meals serves to decrease the demand of unconsumed food [49]. This also mirrors the results of a previous study wherein larger households are found to be more efficient in meal production [50]. On the other hand, fish, meat, and poultry plate waste was less likely in households with ≤ 5 members than in households with more than 5 members.

Regarding wealth quintile, households belonging to the richest quintile were found to have greater plate waste compared to the poorest quintile. This corresponds with the findings of a previous study wherein higher-income households were found to waste more food than lower-income households, which may be explained that higher-income households consume diets that tend to include more perishable items, some of the waste can be explained by food spoiling before the household had a chance to eat it [51]. Moreover, according to the FAO report, the most important reason for food waste at the consumption level among richer individuals is that people simply can afford to waste food [52].

Millions of Filipinos under poverty and experiencing food insecurity are struggling to be fed, and the food that is simply thrown away or discarded might actually be enough to feed them. Plate waste also generally emits a portion of the total global greenhouse gas emissions that cause on impact on global warming [5]. Hence, our results reinforce the need for new strategies to focus on reducing plate waste, which is beneficial from a nutritional, economic, and environmental point of view. In general, the study suggests a more effective strategy for reducing food waste may be to train people to prepare and select less food (portion and meal size reduction) and to formulate more policies tackling waste-reduction programs. Both public and private sectors have a role to play to address global food shortages and food wastes considering that more people are getting hungry globally despite available sufficient food for everyone [53].

### Limitations of the study

The present study has limitations. The 2018 ENNS did not capture the reasons for plate waste, lacking a more depth understanding of the plate waste behavior, as well as the individual or average amount of plate waste per age group.

## Conclusions

This is the first study to explore the association between household dietary consumption andplate waste. Plate waste was significantly higher among food secure households compared to other categories. Our results suggest that rice, vegetable, and meat are the common major food in plate waste. The attributable factors of rice, vegetable and meat plate wastage are larger household meal portion size, a greater number of household members, and higher wealth status. Findings suggest that plate waste is indeed a public health problem that should be addressed. Future research studies should explore the nutrient losses that might stem from plate wastage in order to have a more accurate approach when it comes to the development of strategies and interventions aimed at reducing household plate waste.

Indeed, plate waste is a public health problem that should be addressed.

## Data Availability

The data that support the findings of this study are available from DOST-FNRI but restrictions apply to the availability of these data, which were used under permission for the current study, and so are not publicly available. Data are however available from the authors upon reasonable request and with signed memorandum of agreement with the DOST-FNRI.
